# P05 Introducing new laboratory equipment for blood culture tests to improve the timeliness of antimicrobial susceptibility results in sepsis patients: a quality improvement project

**DOI:** 10.1093/jacamr/dlad066.009

**Published:** 2023-06-14

**Authors:** Rachel Paul, Rajesh Rajendran, Simon Eccles, Michelle Worsley

**Affiliations:** Manchester University NHS Foundation Trust, Infection Prevention and Control Team and University of Salford, UK; Manchester University NHS Foundation Trust, Infection Prevention and Control Team and University of Salford, UK; Manchester University NHS Foundation Trust, Infection Prevention and Control Team and University of Salford, UK; Manchester University NHS Foundation Trust, Infection Prevention and Control Team and University of Salford, UK

## Abstract

**Background:**

Blood culture tests are crucial for identifying microorganisms causing sepsis and planning targeted antimicrobial therapy based on susceptibility results. However, the traditional testing method has remained unchanged for the last 60 years, resulting in a delay of approximately four days for final antimicrobial susceptibility results. This delay contributes to increased antimicrobial resistance, broad-spectrum antimicrobial use and healthcare costs. This project aims to reduce the time taken for antimicrobial susceptibility results.

**Methods:**

An initial pilot study trial of 100 samples with a new system will be conducted, followed by a qualitative and quantitative evaluation strategy, keeping the existing system as a gold standard. The project scope was defined to avoid scope creep, and a detailed SWOT analysis was conducted to identify the best equipment available on the market.

**Interventions:**

A process map was created, and a detailed fish bone (Ishikawa) problem analysis was done to identify key issues and potential bottlenecks. A robust stakeholder analysis has been planned, and a detailed GANTT chart has been prepared. Implementing effective communication, leadership and nudge theory can engage stakeholders and mitigate risks. A cost-benefit analysis predicted a cost savings of nearly a million pounds within one hospital of seven within the Trust.

**Conclusions:**

The introduction of new laboratory equipment for blood culture tests has the potential to reduce the turnaround time for antimicrobial susceptibility results significantly to less than 24 h, leading to early diagnosis and treatment of sepsis with targeted antimicrobial therapy. This initiative also counters various problems, including antimicrobial resistance, broad-spectrum antimicrobial use and healthcare costs. This project proposal is an important step towards improving blood culture testing and the timeliness of antimicrobial susceptibility results. Organizations like the NHS must seek to improve continuously for long-term effectiveness, and this project is one such change that will reap many long-term benefits.

